# Analysis of Bonding Slip Performance Between Novel Epoxy Resin Concrete and Reinforcement

**DOI:** 10.3390/ma19214580

**Published:** 2026-10-29

**Authors:** Jingwei Cai, Shengwei Yin, Yi Zhu, Yanyan Liu, Xinsheng Yin, Guanjie Li

**Affiliations:** 1 Key Laboratory Comprehensive Energy Saving of Cold Regions Architecture of Ministry of Education, Jilin Jianzhu University, Changchun 130119, China; 2 School of Civil Engineering, Jilin Jianzhu University, Changchun 130119, China; yinswei2001@163.com (S.Y.);; 3 National Engineering Research Center of Biomaterials, Nanjing Forestry University, Nanjing 210037, China; 4 Changchun Urban Construction and Maintenance Group Co., Ltd., Changchun 130062, China

**Keywords:** epoxy resin concrete, reinforcement, bond–slip behavior, pull-out test, anchorage length

## Abstract

This study systematically investigated the bonding behavior between novel epoxy resin concrete (ERC) and ribbed HRB400 steel bars through central pull-out tests. A total of 30 groups of specimens were designed to evaluate the effects of concrete strength, concrete cover thickness, bar diameter, anchorage length, and stirrup ratio on bond performance. The results indicate that increasing ERC strength significantly enhances the bond with reinforcement, primarily by improving chemical adhesion and mechanical interlock, while inhibiting the development of internal cracks. Concrete cover thickness was found to have a significant impact on bond strength; increasing the cover within a reasonable range effectively prevented splitting failure. Enlarging the bar diameter from 16 mm to 20 mm improved bond strength, but the benefit diminished beyond 20 mm. A negative correlation was observed between anchorage length and ultimate bond strength. Bond stress was unevenly distributed along the anchorage length, with higher stresses at both ends and lower stress in the middle region. Compared with conventional concrete of equivalent grade, the bond performance between ERC and steel bars was improved by 79%. These findings provide a theoretical basis for the design and application of ERC in engineering structures.

## 1. Introduction

Epoxy resin concrete (ERC) is a kind of advanced concrete material that offers several advantages, including high strength, good toughness, impact resistance, corrosion resistance, impermeability, and freeze–thaw durability [1]. One of the key issues in the structural design of such materials is the bond performance between ERC and steel reinforcement. Studies by Qian Yongmei et al. [2] have shown that the tensile strength of ERC under specific conditions is significantly higher than that of conventional concrete of the same grade. According to the concrete structure design code GB 50010-2002 [3], the bond–slip strength between concrete and reinforcement is positively correlated with the tensile strength of the concrete [4].

A limited number of systematic studies have been conducted on the bond behavior between steel bars and ERC [5]. Therefore, this study designed and fabricated 24 sets of ERC specimens with HRB400 ribbed steel bars for bond anchorage tests. Using central pull-out, the effects of various parameters, such as ERC strength, concrete cover thickness, anchorage length, bar diameter, and stirrup ratio, on bond performance were investigated [6]. Based on the experimental results, a simplified calculation formula for the anchorage length of reinforcing bars in new epoxy resin concrete and a representation of the local bond stress distribution along the anchorage length were proposed, providing an explanation for the anchorage length effect. The results provide a theoretical basis for the application and design of ERC in engineering structures.

## 2. Experimental Overview

### 2.1. Reinforcement Steel

The reinforcing steel used in this experiment is HRB400 steel; its specific measured parameters are shown in Table 1.

### 2.2. Novel Epoxy Resin Concrete

The concrete materials used in this experiment are classified into two types: novel ERC and a low-epoxy resin content variant of the novel epoxy resin concrete (L-ERC). The compressive strengths of these two materials correspond to the C50 and C30 grades of ordinary concrete, respectively. Both materials are composed of oily epoxy resin, fine sand, hardener, anhydrous ethanol, and fly ash. After preheating and mixing, the mixture was poured into molds and compacted. Once the specimens formed, they were placed in a cool, dry environment to cure for 7 days. The specific mix ratios and material physics for ERC and L-ERC are shown in Table 2.

### 2.3. Specimen Design

**Figure 1:**
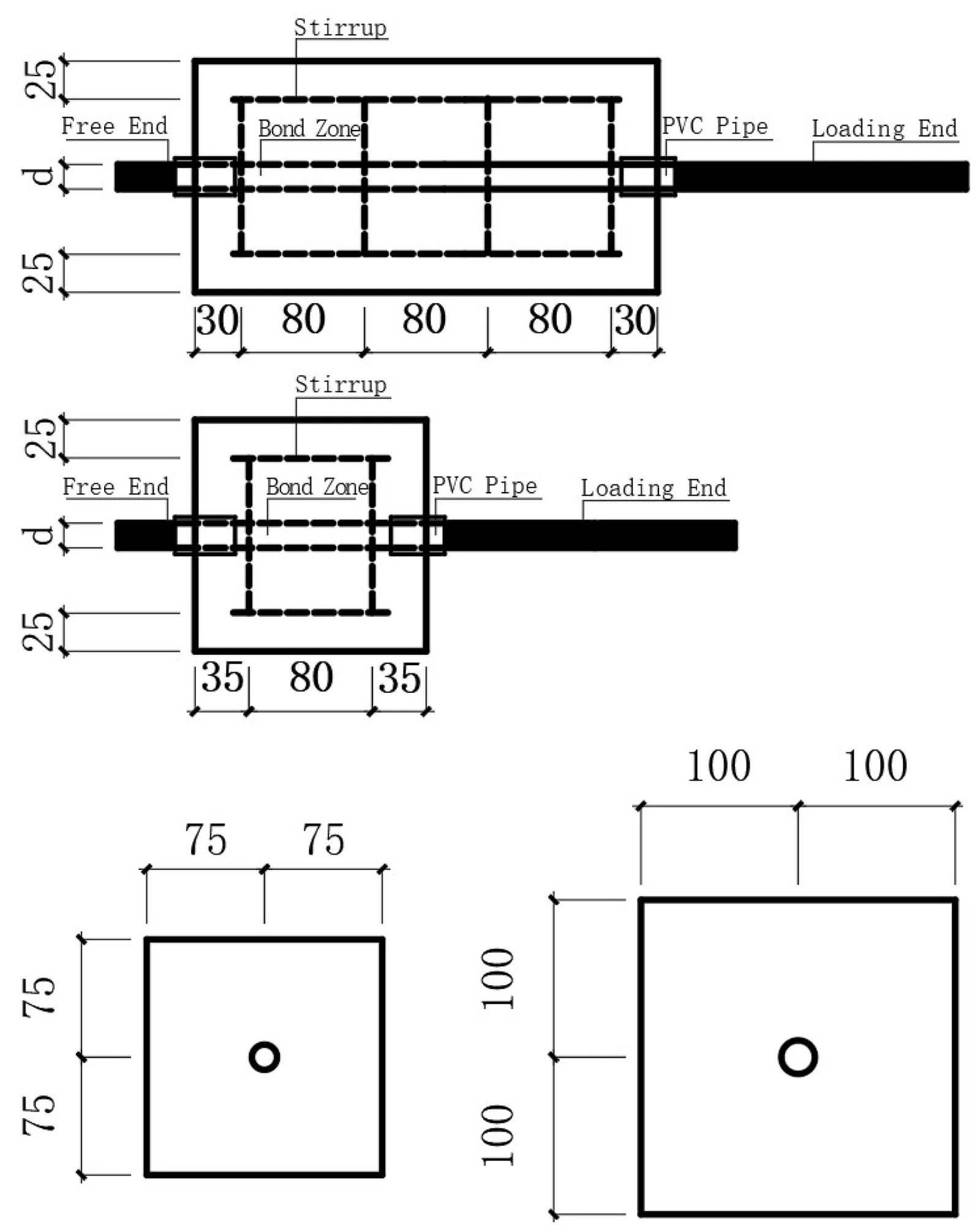
The size of the specimens.

The specimens were designed in three specifications (150 mm × 150 mm × 150 mm, 150 mm × 150 mm × 300 mm, and 200 mm × 200 mm × 200 mm), with a total of 30 experimental groups. The specific parameters are outlined in Table 3. The strength levels of the novel epoxy resin concrete are classified into two categories: ERC and L-ERC. For the reference specimens, the effective anchorage length was set at 5d. Based on the dimensions of the loading apparatus, the length of the reinforcing bars extending beyond the concrete surface at the loading end was set to 200 mm, while the free end extended 50 mm beyond the concrete surface. In reference to the studies of Yuan Jian et al. [3] and Hao Tong et al. [6] on the effect of stirrups on bond performance, stirrups were placed within most of the specimens to more accurately measure the ultimate bond strength. The basic specimen design is illustrated in Figure 1.

**Figure 2:**
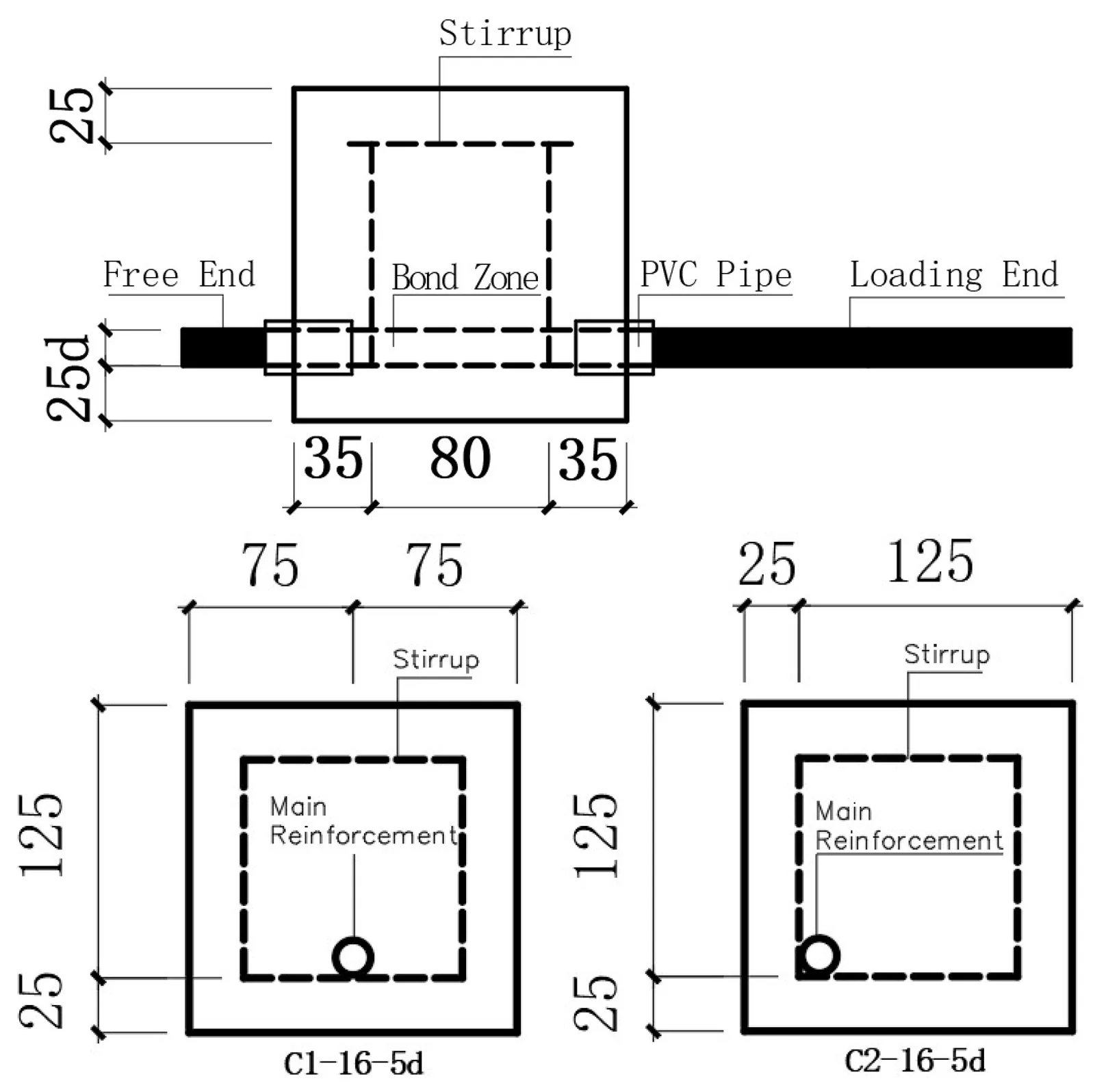
Eccentric reinforcement diagram.

To investigate the effect of concrete cover thickness on bond strength, this experiment altered the cover thickness using eccentric reinforcement placement. As shown in Figure 2, two different reinforcement placement methods were employed to compare the influence of cover thickness on bond strength.

**Figure 3:**
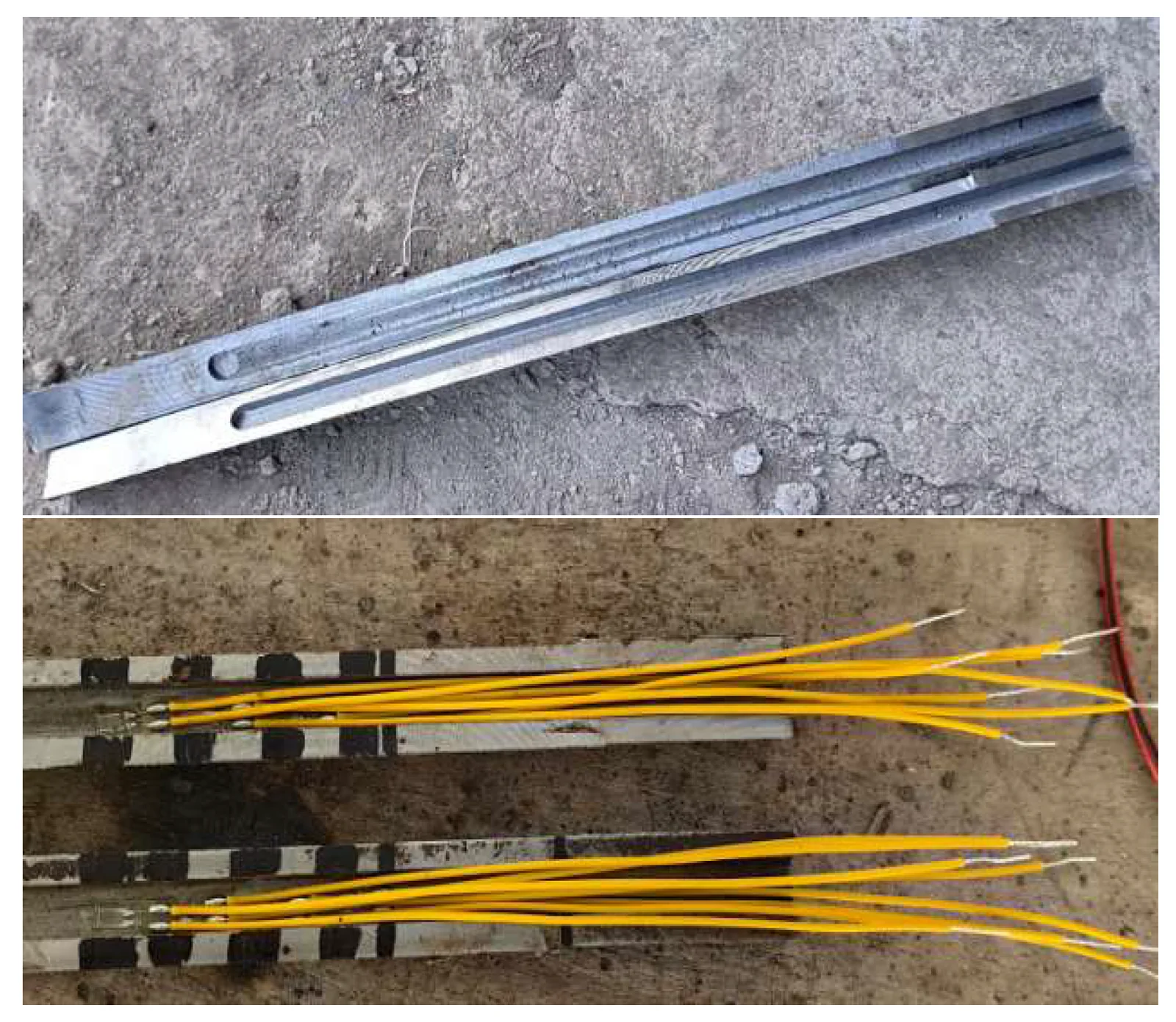
Strain gauge layout diagram.

Since the data obtained from the pullout test represents the average bond stress during the pullout process and the relative slip between the reinforcement and concrete, it does not account for the variation in bond stress at different locations along the reinforcing bar [7]. To examine the variation in bond stress along the embedded length of the reinforcement, this experiment used strain gauges attached to the reinforcement to observe the stress changes at different positions along the bond zone. The attachment method is shown in Figure 3. The reinforcement was milled to half its thickness, and a groove was cut in the middle, with dimensions of 2 mm × 8 mm. Strain gauges were then evenly spaced at 2 mm intervals along the groove, and the positions of the gauges were marked and recorded. For the E and F series specimens, the reinforcing bar was locally machined to half of its original thickness in the short central gauge segment, and a 2 mm × 8 mm longitudinal groove was cut to accommodate strain gauges. Outside this short instrumented segment, the bar retained its full cross-section; consequently, the overall axial stiffness of the member remained essentially unchanged compared with an un-machined bar. This ensures that the measured strain distribution reliably reflects the real behaviour of the intact reinforcement.

### 2.4. Experimental Method

**Figure 4:**
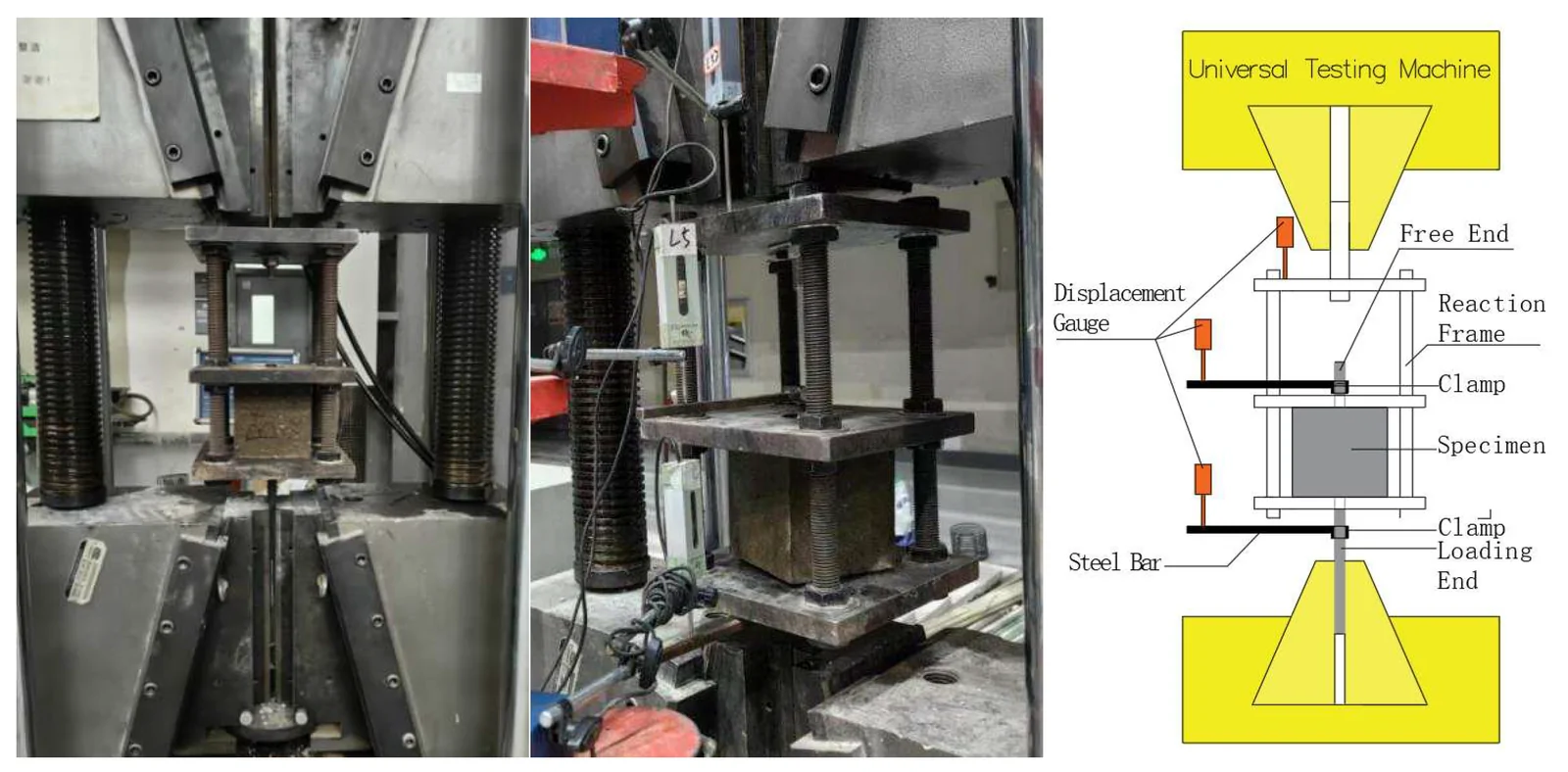
Test setup.

In this experiment, a 200-ton universal tensile-compressive testing machine was used to perform unidirectional loading on the specimens, which were placed within a reaction frame. The specific test setup is shown in Figure 4. Since the elongation of the loaded reinforcement is a combination of its own deformation and the slip displacement, to accurately measure the pure slip, elongation fixtures were installed at both ends of the reinforcement. Displacement gauges were placed at the loading end, free end, and the entire reaction frame to measure the displacement, from which the relative slip between the reinforcement and concrete can be calculated.

After obtaining the measured data, the relative slip *s* and bond strength *τ* between the reinforcement and concrete at various load levels *F* were calculated using Equations (1) and (2) [8,9,10].

(1) $$s_{L}={\;s}_{d}\;-{\;s}_{b}=s_{d}\;-\;{\frac{1}{2}(s_{1}}_{⁠}+{\;s}_{2})\;-\;\frac{\mathrm{FL}}{\mathrm{EA}}$$

(2) $$Τ=\frac{F}{\pi dl_{a}}$$

In the equations, *S_L_* represents the relative slip value; *S_d_* is the total slip of the reaction frame; *S_b_* is the elongation of the reinforcement; *S*_1_ and *S*_2_ are the displacements at the loading end and free end, respectively; *F* is the applied load; *L* is the distance from the bond initiation point at the loading end to the testing machine reference point; *E* is the elastic modulus of the reinforcement; and *A* is the cross-sectional area of the loaded reinforcement.

Loading was stopped and the test was considered complete when any of the following conditions occurred: (1) the concrete undergoes splitting failure; (2) the reinforcement fractures; (3) the testing machine shows a significant loss of load, the load–displacement curve becomes stable, and the reinforcement is pulled out.

## 3. Experimental Phenomena

The failure modes of the experiment are classified into pullout failure, splitting-pullout failure, and reinforcement yield failure [11].

### 3.1. Pullout Failure

**Figure 5:**
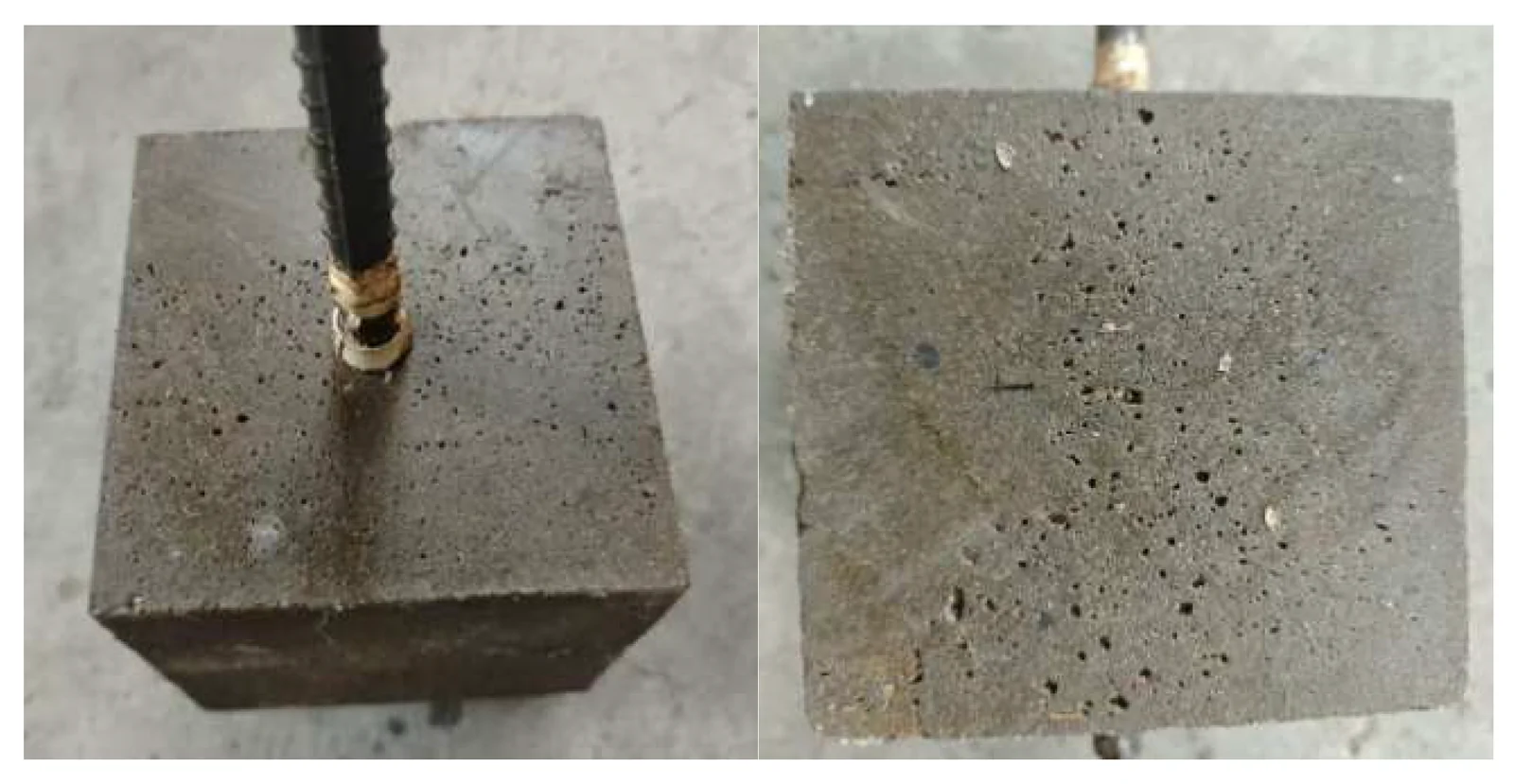
Pull-out failure.

As shown in Figure 5, pullout failure occurs when the concrete cover is sufficient or when stirrups are provided, which offer strong lateral confinement to the reinforcing bars. Due to the shear effects generated by the transverse ribs of the reinforcement on the surrounding concrete, cracks are initiated within the concrete [12,13]. However, before these cracks propagate to the surface, the concrete between the ribs is crushed, losing its confinement. As a result, the reinforcement is directly pulled out, leading to pullout failure. During this failure mode, no obvious surface cracks are observed on the specimen.

### 3.2. Splitting-Pullout Failure

**Figure 6:**
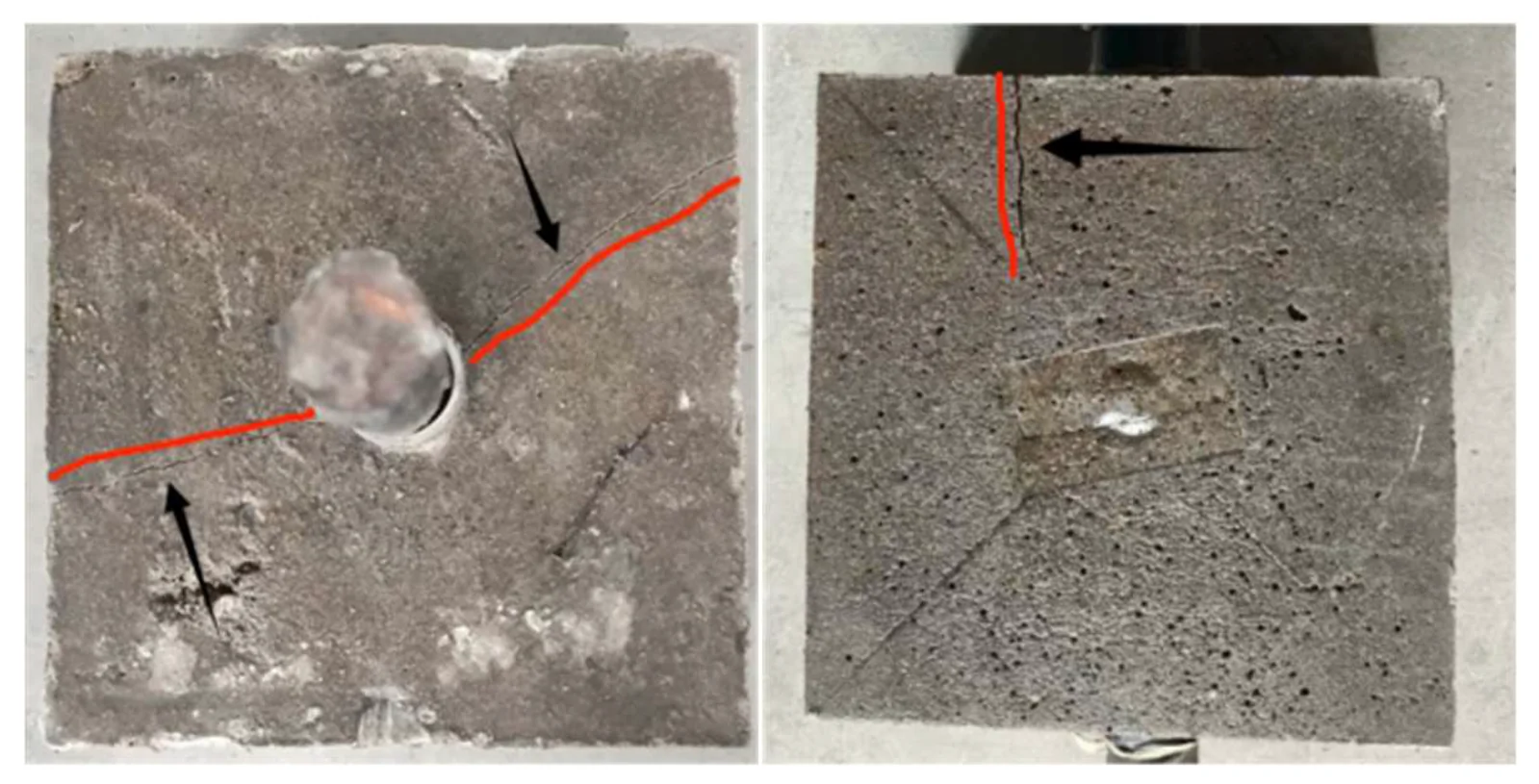
Splitting-pull-out failure.

As shown in Figure 6, splitting-pullout failure occurs when the concrete cover is of moderate thickness and stirrups are provided. The shear effects of the reinforcement’s transverse ribs on the surrounding concrete cause cracks to develop, which eventually propagate to the surface of the concrete. This leads to concrete splitting, resulting in a rapid loss of bond strength between the reinforcement and the concrete [14]. Consequently, the reinforcement is pulled out, and visible cracks appear on the surface of the concrete.

### 3.3. Reinforcement Yield Failure Bond-Induced Rebar Yield Failure

**Figure 7:**
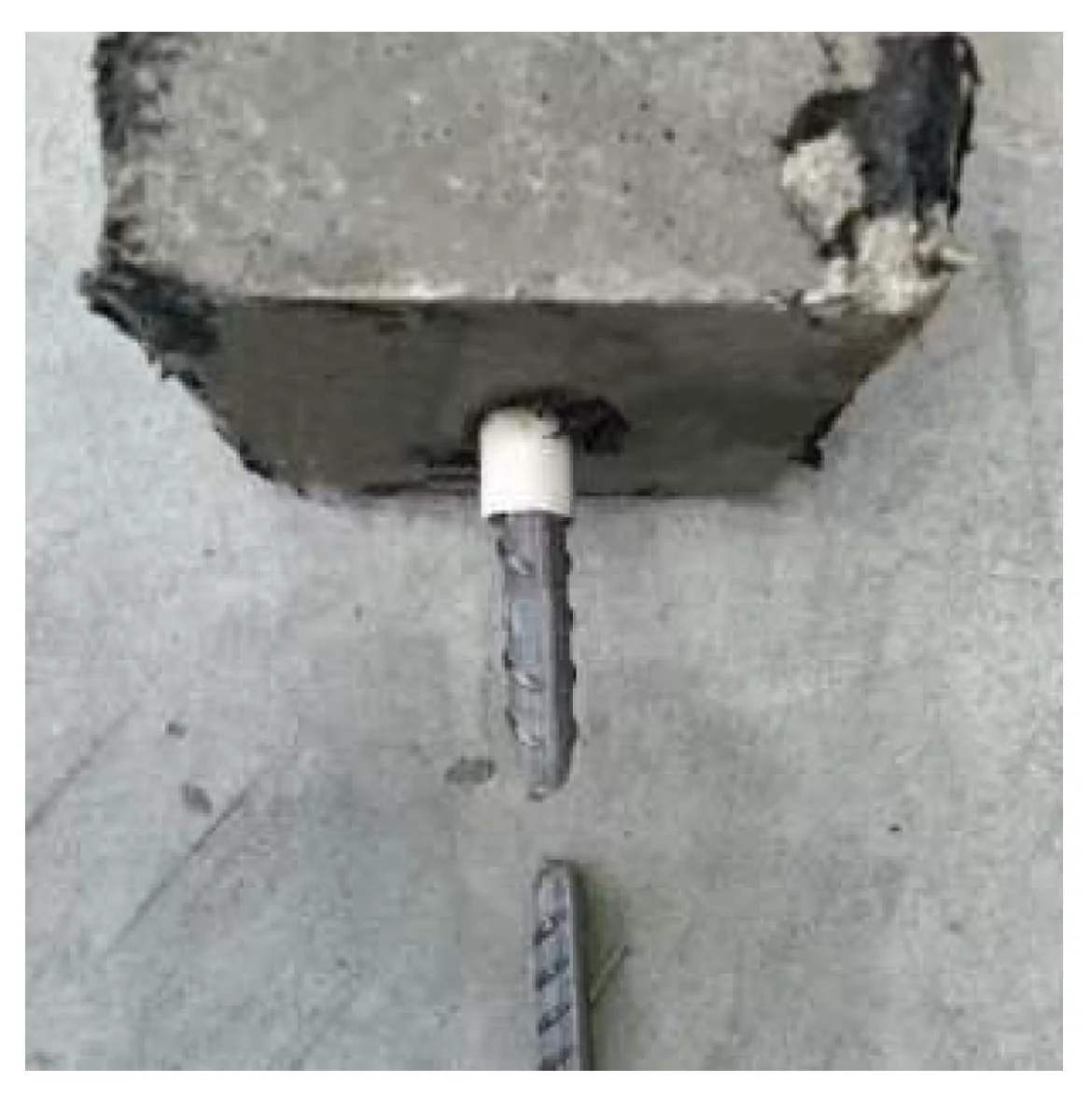
Reinforcement yield failure.

As shown in Figure 7, reinforcement yield occurs when the anchorage length is excessively long, causing the bond between the concrete and the reinforcement to be too strong. During the pullout process, the failure occurs at the mid-point between the testing machine reference point and the bond initiation point, resulting in the yield of the reinforcement. The reinforcement is not pulled out, and no visible cracks appear on the surface of the concrete.

## 4. Experimental Results

### 4.1. Bonding Performance of Ribbed Steel Bars with Novel ERC

Table 4 presents the average compressive strength of cubes under the same curing conditions (*f_cu_*), the average splitting tensile strength (*f_tu_*), the average ultimate load (*F*), the average ultimate bond strength (*τ_u_*), the average relative slip between the steel bar and concrete at ultimate load (*S*), as well as the observed failure modes. In the failure modes, *B* denotes pull-out failure, *S* denotes splitting–pull-out failure, and Y denotes steel yielding failure.

### 4.2. Influence of Concrete Strength

**Figure 8:**
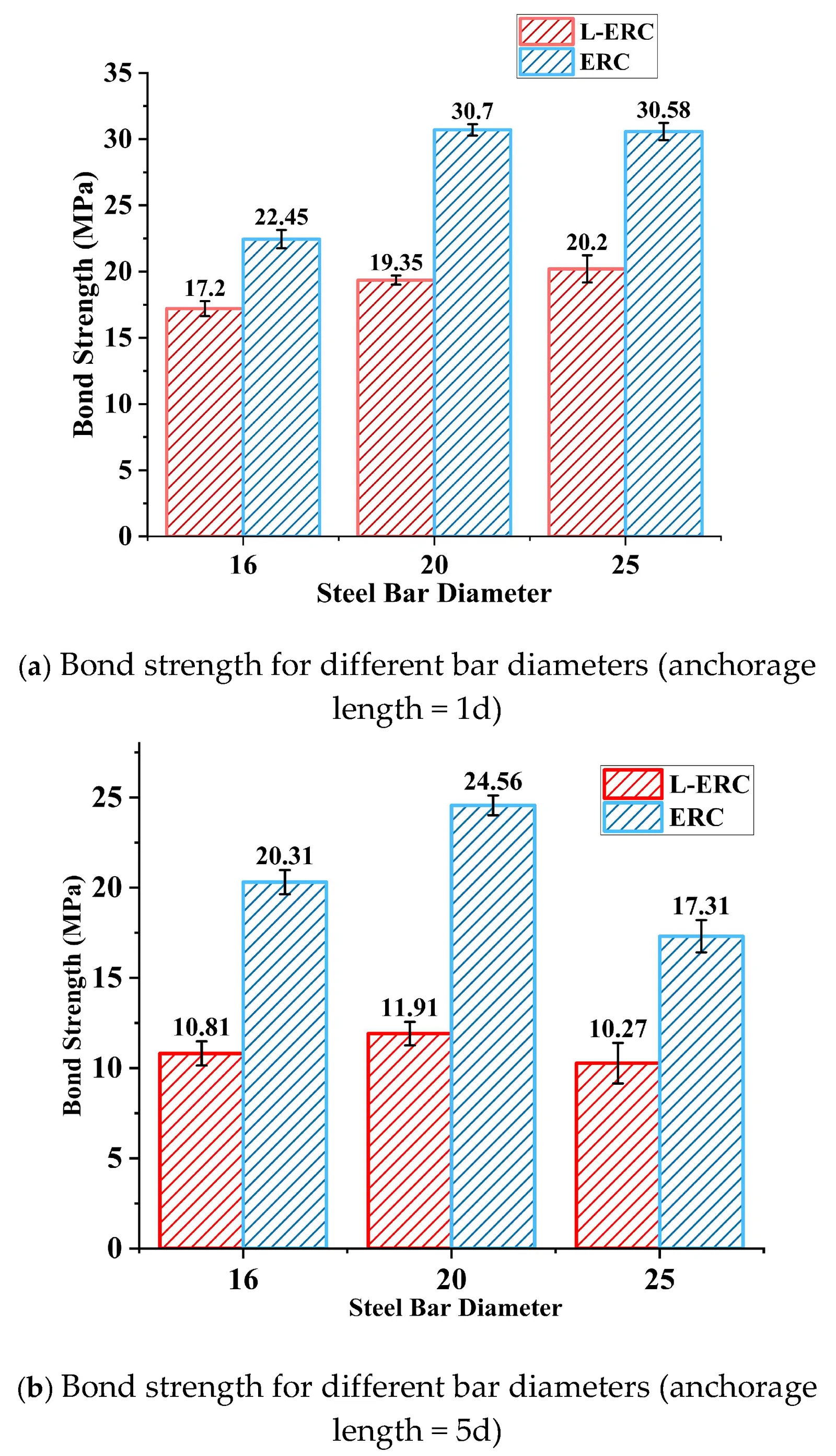
Influence of concrete strength on bond stress. Bond strength for different bar diameters.

As shown in Figure 8, the experimental groups with anchorage lengths of 1d and 5d were selected for comparison. Under otherwise identical conditions, the enhanced compressive and tensile strengths of the novel epoxy resin concrete significantly improved the ultimate bond strength of the specimens. Compared to conventional concrete of the same compressive strength grade, the epoxy resin concrete exhibited a notably higher tensile strength.

During the loading process, the bond behavior is primarily governed by chemical adhesion, frictional resistance, and mechanical interlocking. The strength enhancement of the epoxy resin concrete markedly increased the chemical bonding and mechanical interlock between the reinforcing steel and the surrounding concrete. Within a certain range, increasing the epoxy resin content not only improves the chemical adhesion and tensile properties of the concrete, but also effectively inhibits the development of internal cracks. This delay in crack propagation helps to mitigate bond degradation caused by the crushing of concrete between the ribs of the reinforcement.

In addition, compared with the tensile strength values of conventional concrete reported by Xu Youlin et al. [8], the epoxy resin concrete demonstrated superior bonding performance under the same tensile strength grade. This is attributed to the fact that epoxy resin concrete is composed entirely of mortar, without coarse aggregate. The absence of large aggregate significantly reduces the longitudinal component of the force transmitted through the transverse ribs of the reinforcement, which helps to slow the growth of internal cracks and thereby enhances the bond behavior between steel and concrete.

### 4.3. The Effect of Lateral Confinement

**Figure 9:**
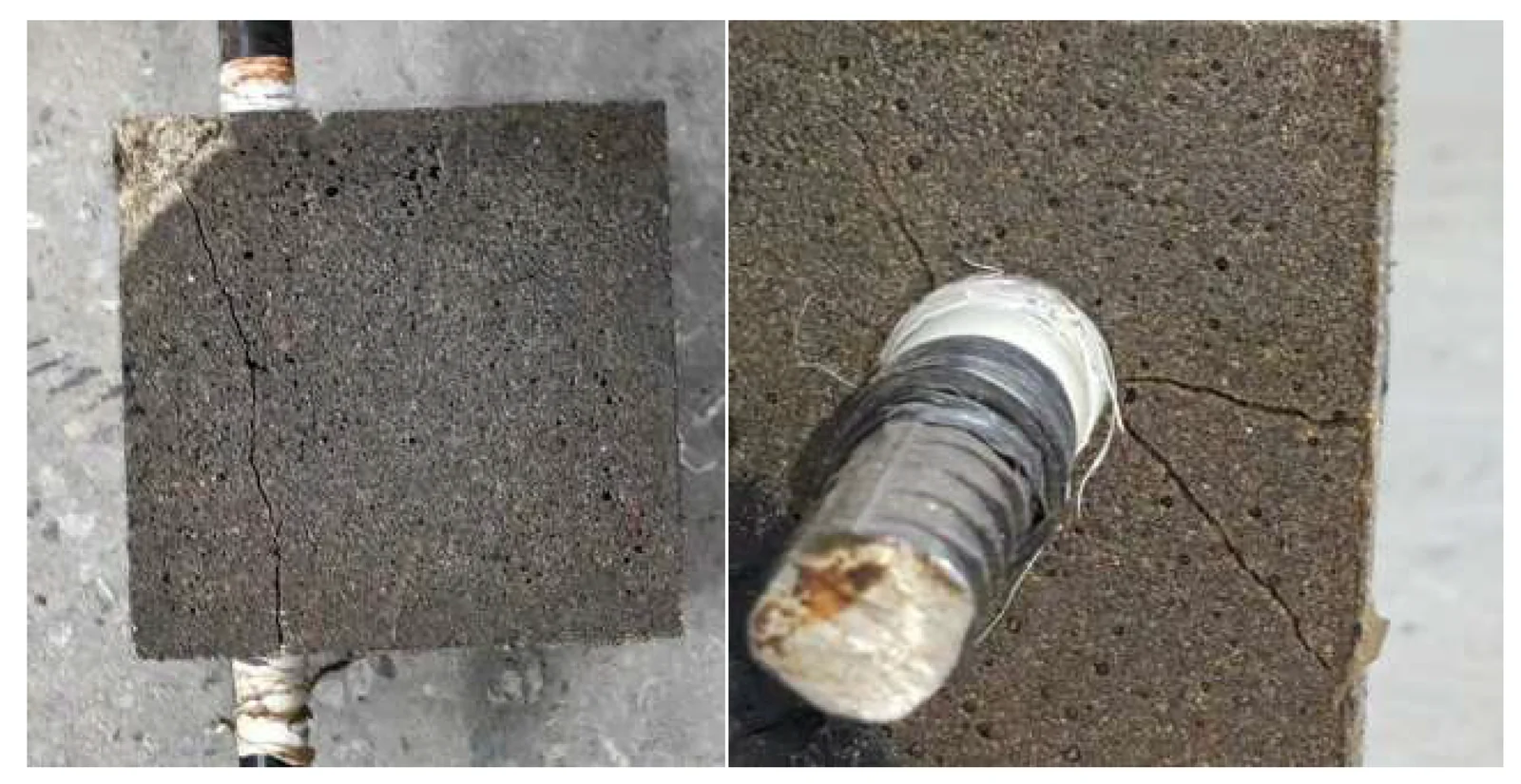
Effect of concrete cover thickness on bond behavior.

The specimens C1-16-5d and C2-16-5d were subjected to two different reinforcement placement methods to investigate the effect of concrete cover thickness on the bond performance. As shown in Figure 9, the two specimens experienced pullout failure and splitting-pullout failure, respectively. When compared with specimen A1-16-5d and other splitting-pullout specimens, C2-16-5d exhibited more noticeable cracks and a longer lateral crack propagation length. The ultimate bond stress decreased by 16.5% and 25.9%, respectively.

These results indicate that under certain conditions, the ultimate bond stress between the new epoxy resin concrete and rebar increases with the increase in cover thickness. This is because specimens with a thinner cover layer exert less lateral force on the stressed rebar, making it more prone to internal cracking. The cracks grow faster and reach the concrete surface more quickly, causing the concrete specimen to split more easily and exhibit more pronounced cracks.

D1-16-5d and D2-20-5d were designed by removing the internal stirrups to study the effect of stirrups on the bond stress. As shown in Table 4, under the same conditions, the ultimate bond stress of the two specimens decreased by 0.01% and 19.0%, respectively, with D2-20-5d experiencing splitting-pullout failure. The average bond strength of D1-16-5d without stirrups was 20.11 MPa, which is only 1.0% lower than the 20.31 MPa measured for the stirrup-confined specimen A1-16-5d. This marginal difference indicates that the presence of stirrups has a negligible influence on the ultimate bond strength for the present test configuration.

C3-25-3d has dimensions of 200 mm × 200 mm × 200 mm and increases the concrete cover thickness around the 25 mm diameter rebar by enlarging the overall specimen size. This specimen underwent pullout failure, and compared with A8-25-3d, which experienced splitting-pullout failure, the ultimate bond stress increased by 39.6%.

These results indicate that the thickness of the concrete cover has a more significant effect on the bond strength between the new epoxy resin concrete and rebar. Therefore, when the concrete cover reaches a certain threshold and prevents splitting-pullout failure, the effect of stirrups on bond stress becomes negligible.

### 4.4. The Impact of Rebar Diameter

**Figure 10:**
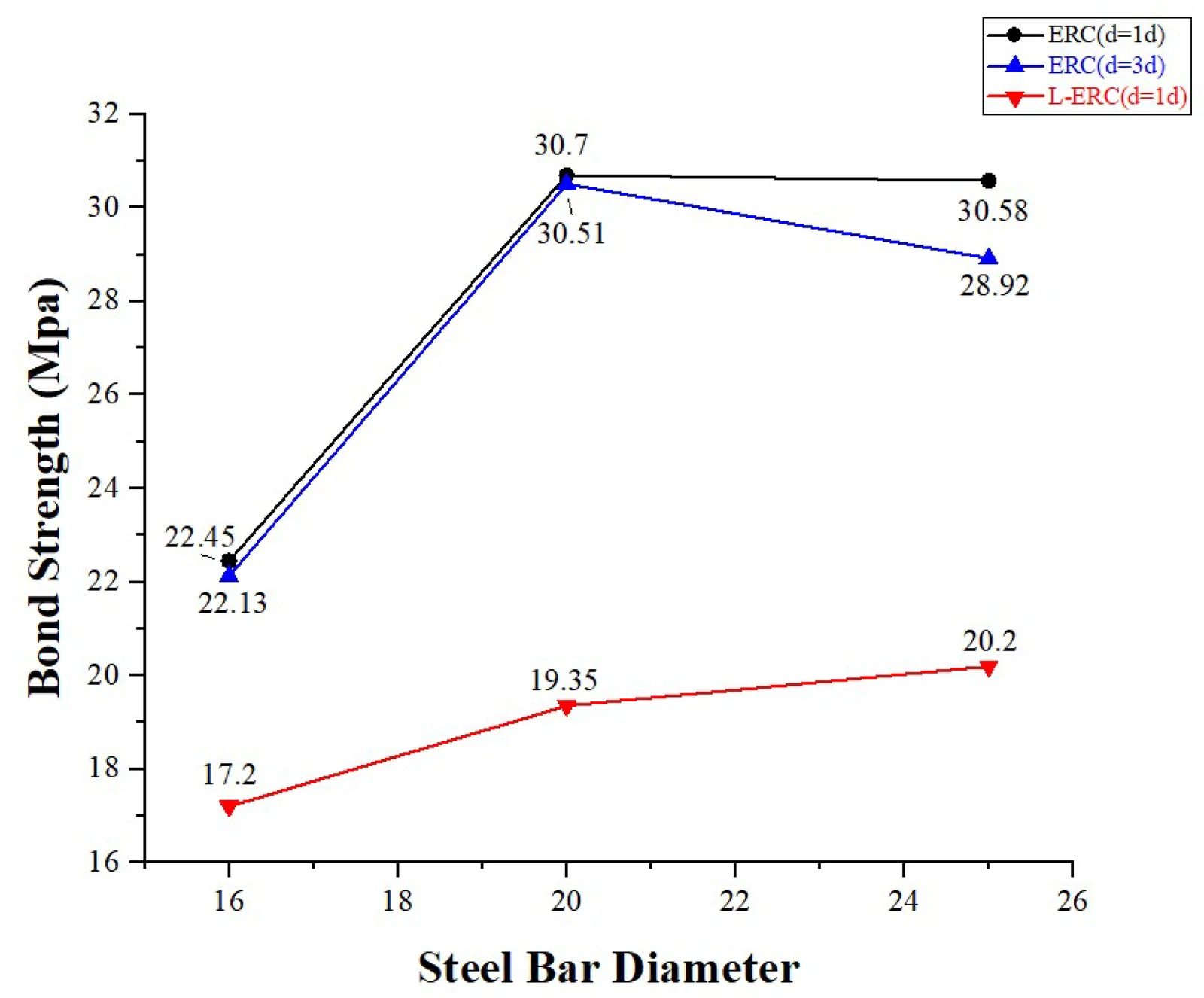
Influence of reinforcement diameter.

As illustrated in Figure 10, to eliminate the interference of concrete splitting failure on the evaluation of ultimate bond strength, three groups of specimens exhibiting pure pull-out failure were selected for comparison. It can be observed that, for the newly developed ERC, the relative bond strength tends to increase with the enlargement of the rebar diameter. However, when the diameter exceeds 20 mm, the increase becomes marginal.

This trend may be attributed to the fact that the mechanical properties of ERC can be more effectively mobilized both the rib between the ribs. Nevertheless, when the rebar diameter surpasses 20 mm, the relative rib area decreases, leading to a diminished contribution to the enhancement of ultimate bond strength.

In addition, previous studies [9,10] have indicated that in conventional concrete, an increase in rebar diameter generally results in either a reduction or negligible change in ultimate bond strength. In contrast, in ERC, increasing the diameter of the tensioned rebar can improve the bond strength by up to 39%. This significant improvement is likely due to the absence of coarse aggregates in ERC, which is essentially a mortar-based composite. This absence substantially reduces the lateral splitting force generated by the transverse ribs of the rebar. Meanwhile, the excellent tensile properties of ERC help suppress the propagation of cracks around the rebar, thereby enhancing the overall bond performance [7,11].

**Figure 11:**
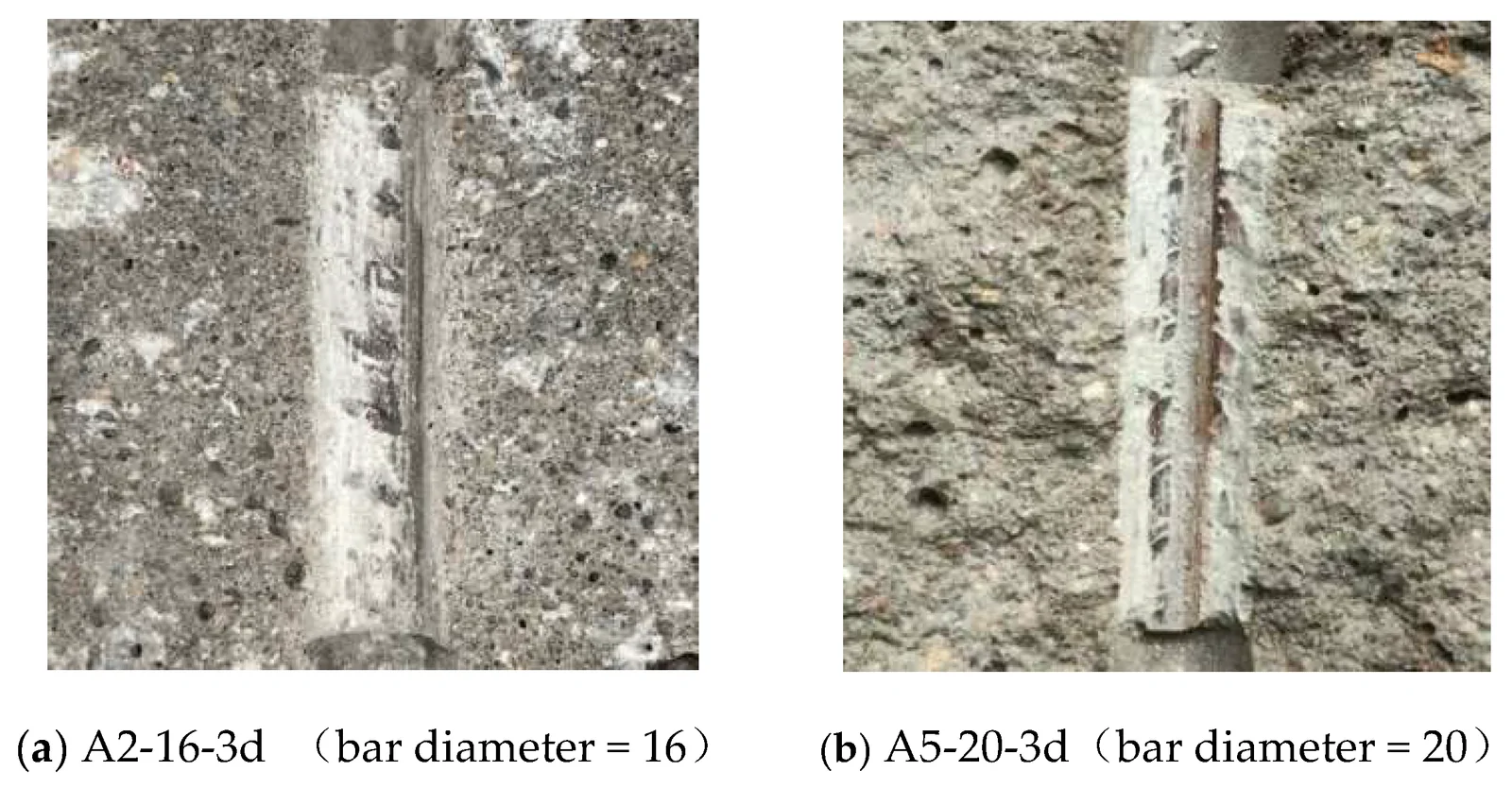
Concrete failure interface in the bond zone for different bar diameters.

As shown in Figure 11, increasing the rebar diameter significantly raises the ultimate load, which intensifies the interaction force between the rebar ribs and the surrounding concrete. This stronger interaction leads to more severe crushing of the concrete within the bond zone, resulting in finer debris and a smoother failure interface. Consequently, the phenomenon of peeling becomes more pronounced. In the 150 mm cube specimens, increasing the bar diameter from 16 mm to 20 mm and 25 mm inevitably reduces the clear cover-to-diameter ratio c/d from approximately 4.19 to 3.25 and 2.50, respectively. The observed variation in bond strength therefore reflects the combined influence of bar diameter and relative confinement, rather than the effect of diameter alone. With the present specimen geometry, these two factors cannot be separated. Accordingly, the results are interpreted here as an overall diameter-and-relative-cover effect, and no claim is made that bond strength increases with bar diameter up to 20 mm independently of confinement. The failure mode of each specimen is also reported because the change in c/d may influence whether failure occurs by pull-out or splitting. A rigorous evaluation of the diameter effect would require specimens in which the clear cover is adjusted proportionally so as to keep c/d constant; this is left for future work.

### 4.5. The Influence of Anchorage Length

**Figure 12:**
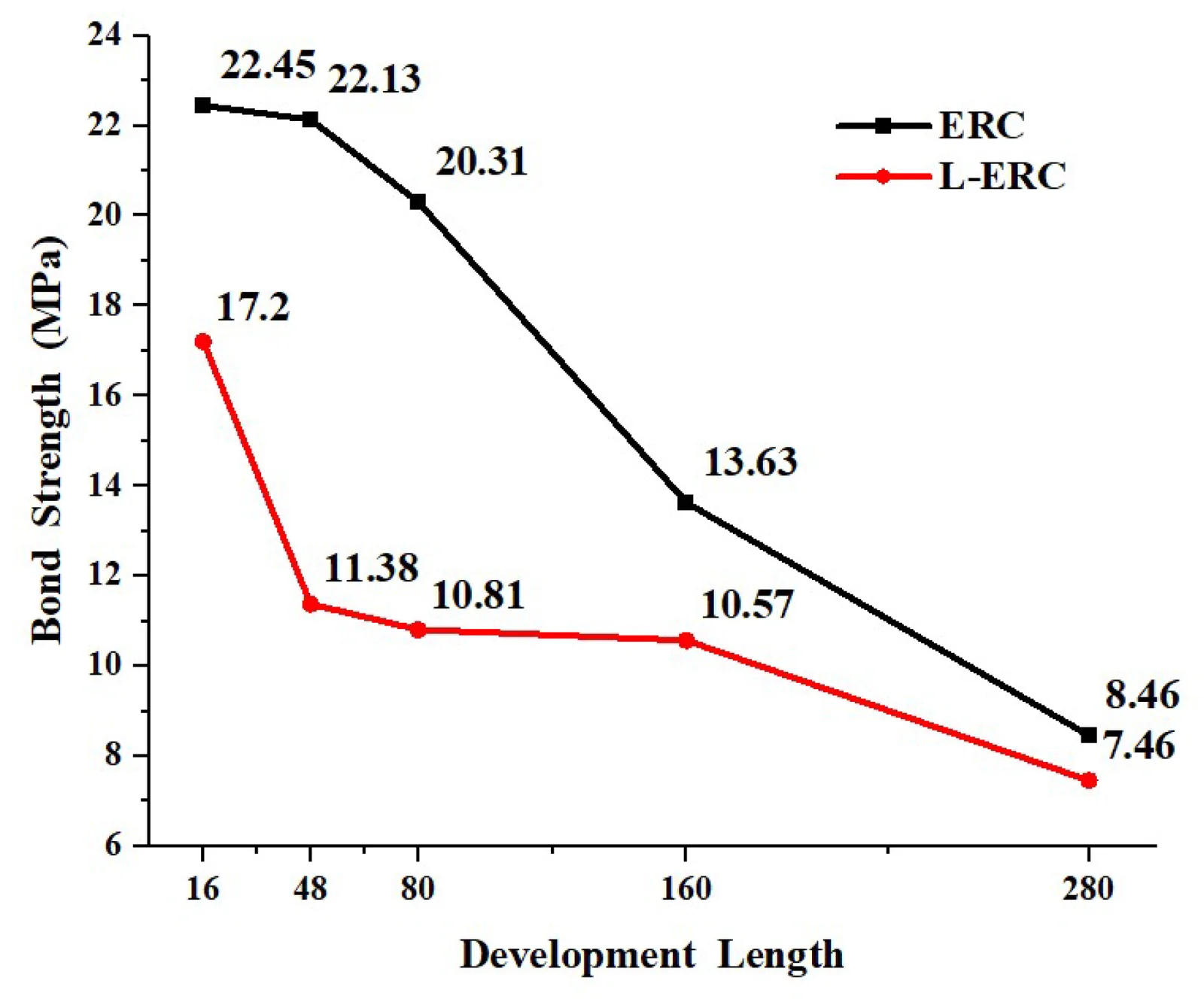
Average ultimate bond stress at different anchorage lengths.

Based on the experimental data, it can be observed that as the anchorage length increases, the average ultimate load of the specimens shows an increasing trend, while the average ultimate bond stress decreases accordingly, as shown in Figure 12.

**Figure 13:**
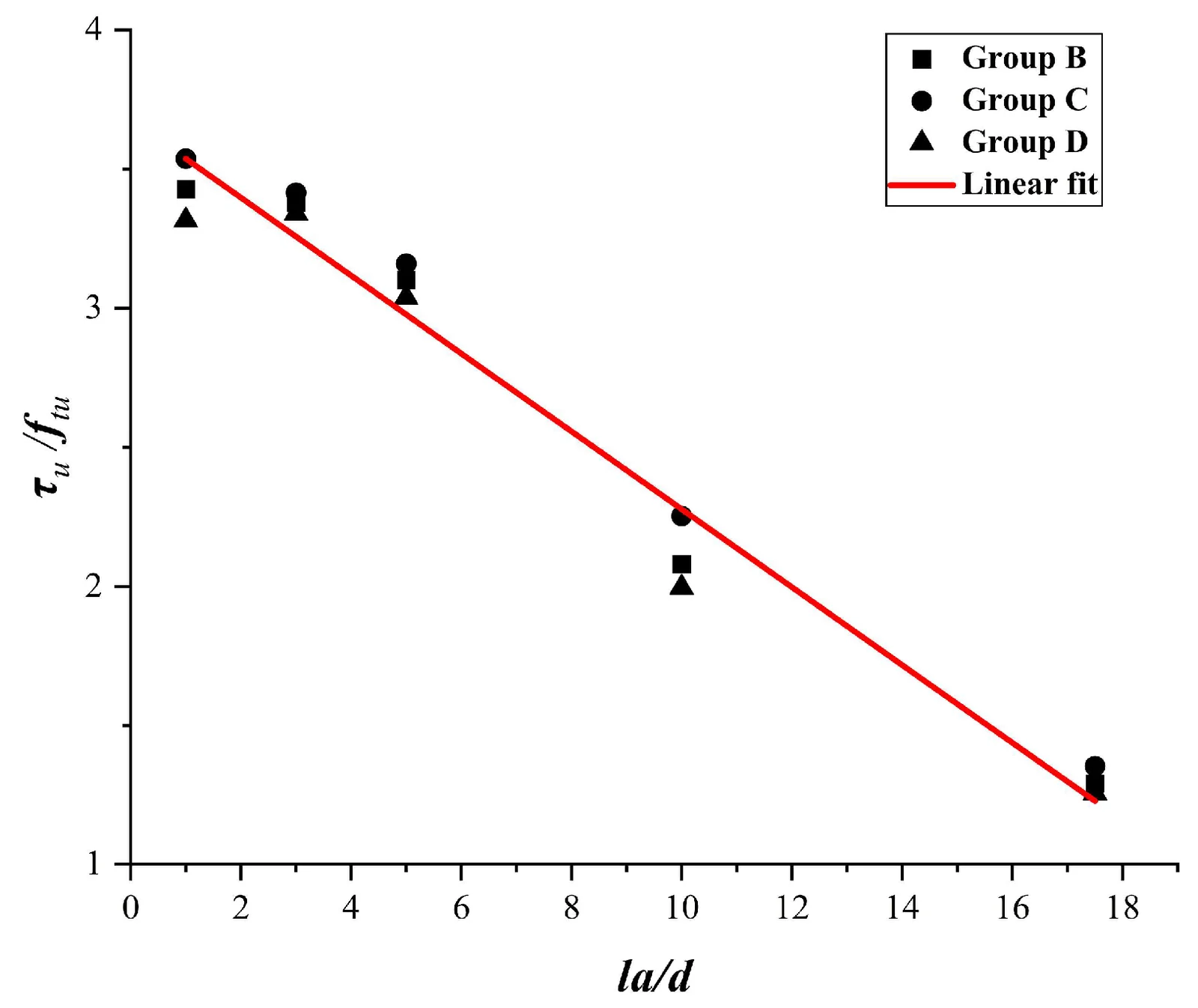
Variation of τu/ftu with la/d for ERC specimens (d = 16 mm).

The data from the ERC specimens with a diameter of 16 were fitted, revealing a nearly linear relationship between the ratio of ultimate bond strength to tensile strength (*τ_u_*/*f_tu_*) and the relative anchorage length (*l_a_*/*d*), as shown in Figure 13 and expressed in Equation (3):

(3) $$\frac{\tau_{u}}{f_{tu}}=3.574-0.08168\frac{l_{a}}{d}$$

Equation (3) should be regarded as a preliminary estimation reference rather than a fully validated design formula. Further pull-out tests over a wider range of bar diameters and concrete strengths are needed to improve statistical confidence and extend the applicability of the proposed equation.

It can be observed that both the ultimate bond strength to tensile strength ratio (*τ_u_*/*f_tu_*) of the novel epoxy resin concrete and the conventional epoxy resin decrease as the relative anchorage length (*l_a_*/*d*) increases. However, when the relative anchorage length (*l_a_*/*d*) reaches or exceeds 5, the value of τ*_u_*/*f_tu_* for the novel epoxy resin concrete does not exhibit the gradual flattening behavior observed in conventional concrete [6]. The novel epoxy resin concrete, with a lower elastic modulus and higher tensile and split-tensile strength, results in a more uniform distribution of bond stress within the material [15].

### 4.6. Distribution of Bond Stress Along the Anchor Length

**Figure 14:**
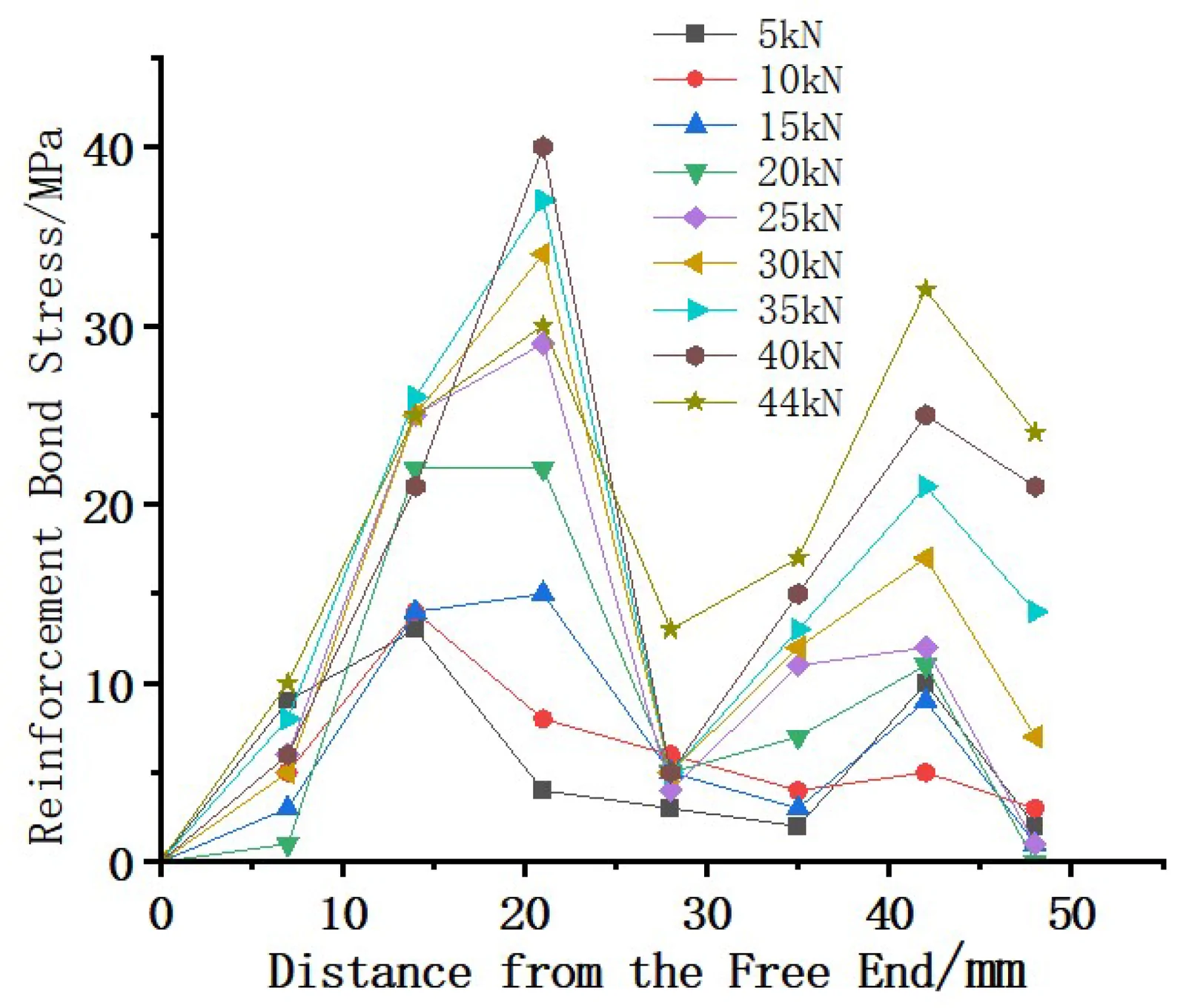
Stress distribution diagram of the specimen with an anchorage length of 48 mm.

The *E* group experiment was conducted to study the variation in bond stress along the length of the rebar anchor. Strain gauges were embedded at equal intervals within the effective anchorage length of the rebar, as shown in Equation (4), to directly measure the microstrain *ε_i_* at specific points on the rebar under loading. At each gauge position, the actual reduced cross-sectional area of the bar was directly measured and substituted into Equation (4) so that the influence of the area reduction was explicitly accounted for in the calculation of local bond stress. This data was then used to calculate the stress level *σ_i_* at each point along the bond length. The bond stress *τ_i_* between two adjacent points was determined based on the variation in stress *σ_i_*. The results are shown in Figure 13 and Figure 14.

(4) $$\tau =\frac{\mathrm{dP}}{\pi d\Delta x}=\frac{d\sigma_{s}A_{s}}{\pi d\Delta x}=\frac{E_{s}A_{s}d\varepsilon_{s}}{\pi d\Delta x}=\frac{E_{s}A_{s}\left(\varepsilon_{i+1}-\varepsilon_{i}\right)}{\pi d\Delta x}$$

**Figure 15:**
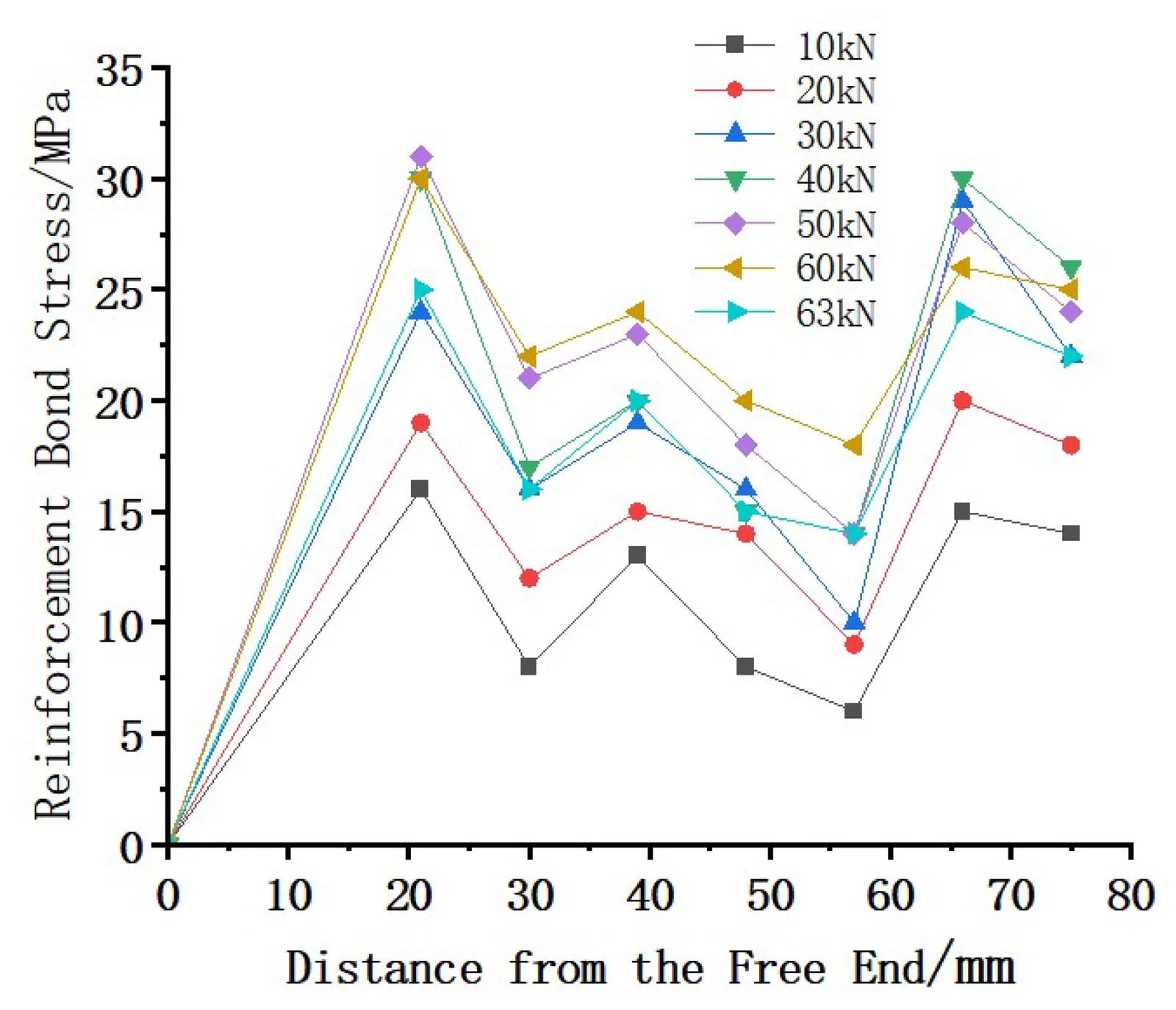
Stress distribution diagram of the specimen with an anchorage length of 80 mm.

As shown in Figure 14 and Figure 15, the bond stress reaches its highest values near the loading and free ends. In specimens with shorter anchorage lengths, the distribution of bond stress is relatively uneven. It is believed that this is due to the new epoxy resin concrete between the ribs of the middle section of the bond zone, which is more prone to fragmentation into powder compared to traditional concrete during the rebar pull-out process, leading to a loss of bond effectiveness. The relative displacement between the concrete and rebar, caused by friction, increases, further fracturing the concrete in the middle section of the bond zone and accumulating it toward the free end, resulting in higher localized stress at the ends. Therefore, the bond stress in the middle section of the bond zone is smaller than that in the sections closer to the loading and free ends.

**Figure 16:**
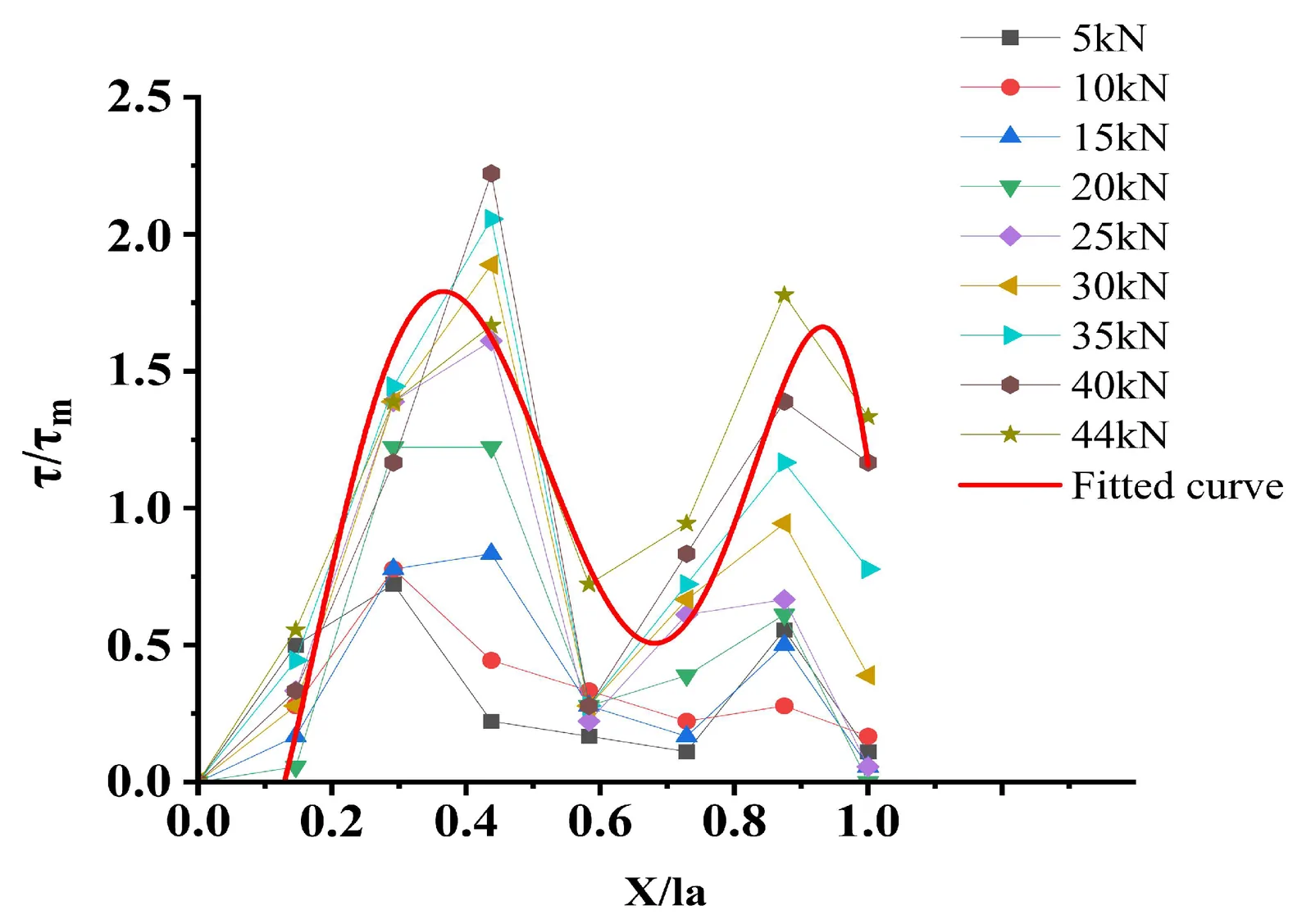
Fitted curve for the specimen with an anchorage length of 48 mm.

**Figure 17:**
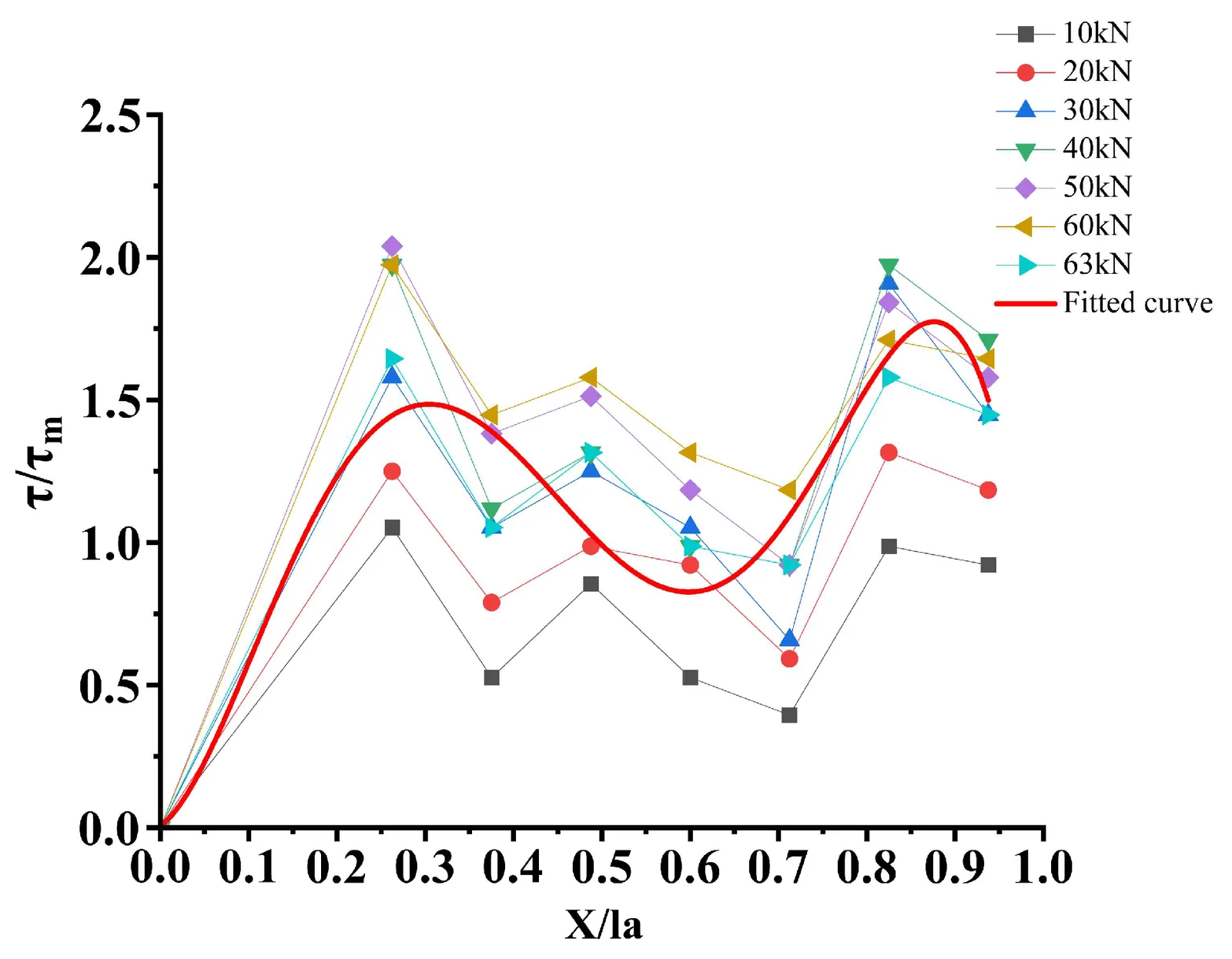
Fitted curve for the specimen with an anchorage length of 80 mm.

By comparing the calculated results with the measured pull-out load and adjusting the bond stress τ_i_ between adjacent measurement points, data fitting was conducted to derive empirical formulas for the bond stress distribution in the bond zone of specimens with different anchorage lengths. The results are shown in Figure 16 and Figure 17, as well as Equations (5) and (6).

(5) $$\tau /\tau_{m}=0.016\;-\;10.498\frac{x}{l_{a}}+114.465{\left(\frac{x}{l_{a}}\right)}^{2}-\;225.202{\left(\frac{x}{l_{a}}\right)}^{3}-\;28.83{\left(\frac{x}{l_{a}}\right)}^{4}+358.445{\left(\frac{x}{l_{a}}\right)}^{5}-\;207.228{\left(\frac{x}{l_{a}}\right)}^{6}$$

The correlation coefficient is 0.76.

(6) $$\tau /\tau_{m}=0.0062+2.196\frac{x}{l_{a}}+57.089{\left(\frac{x}{l_{a}}\right)}^{2}-\;251.237{\left(\frac{x}{l_{a}}\right)}^{3}+352.327{\left(\frac{x}{l_{a}}\right)}^{4}-\;160.03{\left(\frac{x}{l_{a}}\right)}^{5}$$

The correlation coefficient is 0.79.

To verify the reliability of the local bond stress distribution, the local bond stresses were integrated along the anchorage length. For the instrumented specimen F1-16-5d, the local bond stress at each gauge position was multiplied by its tributary surface area, which is $F=\sum_{i=1}^{n}\;\tau_{i}\cdot u\cdot \Delta x_{i},$ and the contributions were summed over the full anchorage length. The resulting total force was then compared with the measured pull-out load. The integrated force was 79.8 kN, close to the measured failure load of 80.83 kN, with a difference of only 1.3%. This good agreement indicates that the strain-gauge measurements and the reduced cross-section correction together produce a consistent force balance along the interface. It also confirms that the local bond stress curves presented in this section are physically reliable and may serve as a sound basis for the mechanistic interpretation of the anchorage-length effect. Formulas (5) and (6) represent the first quantitative empirical description of the local bonding stress distribution in the ERC.

## 5. Conclusions

(1) The enhancement of ERC strength significantly improves its bond performance with steel reinforcement. Compared to ordinary concrete of the same grade and ERC with a low epoxy resin content, ERC can effectively increase the ultimate bond strength by enhancing both chemical bonding and mechanical interlocking. Simultaneously, it suppresses the development of internal cracks, reducing bond performance degradation.
(2) Lateral confinement conditions are a critical factor influencing the bond strength between ERC and steel reinforcement, with the thickness of the concrete cover being particularly influential. When the stirrup ratio is satisfied, increasing the cover thickness can significantly enhance the ultimate bond strength and reduce the risk of splitting failure.
(3) Compared to conventional concrete, increasing the diameter of the reinforcement between 16 mm and 20 mm helps improve bond strength. However, when the diameter exceeds 20 mm, the improvement in bond strength tends to level off.
(4) The anchorage length is negatively correlated with the ultimate bond stress between the ERC and steel reinforcement. As the anchorage length increases, the ultimate bond stress gradually decreases. The ratio of bond strength to tensile strength remains relatively stable as the relative anchorage length changes.
(5) The distribution of bond stress between ERC and the steel reinforcement is uneven. The bond stress is higher near the loading and free ends, while the bond stress in the middle section is relatively lower.

## Figures and Tables

**Table 1:** Mechanical indexes of reinforcement bar.

Steel Bar Diameter	Yield Strength (MPa)	Ultimate Strength (MPa)	Elastic Modulus (GPa)
Φ16	431	577	195
Φ20	463	569	194
Φ25	460	573	194

**Table 2:** ERC and L-ERC mix ratios and material physics.

Type	Epoxy Resin (kg)	River Sand (kg)	Curing Agent (kg)	Anhydrous Ethanol (kg)	Fly Ash (kg)	Average Tensile Strength (MPa)	Average Compressive Strength (MPa)
ERC	434	1580	108	44	40	6.55	53.26
L-ERC	296	1580	177	40	60	3.68	32.58

**Table 3:** Specimen parameters.

Specimen Number	Concrete Type	Steel Bar Diameter *d* (mm)	Anchorage Length *L_a_* (mm)	Cover Thickness (mm)	Steel Bar Diameter and Spacing	Number of Specimens
A1-16-5d	ERC	16	80	67	6@80	3
A2-16-3d	ERC	16	48	67	6@80	3
A3-16-1d	ERC	16	16	67	6@80	3
A4-20-5d	ERC	20	100	65	6@80	3
A5-20-3d	ERC	20	60	65	6@80	3
A6-20-1d	ERC	20	20	65	6@80	3
A7-25-5d	ERC	25	125	62.5	6@80	3
A8-25-3d	ERC	25	75	62.5	6@80	3
A9-25-1d	ERC	25	25	62.5	6@80	3
B1-16-5d	L-ERC	16	80	67	6@80	3
B2-16-3d	L-ERC	16	48	67	6@80	3
B3-16-1d	L-ERC	16	16	67	6@80	3
B4-20-5d	L-ERC	20	100	65	6@80	3
B5-20-3d	L-ERC	20	60	65	6@80	3
B6-20-1d	L-ERC	20	20	65	6@80	3
B7-25-5d	L-ERC	25	125	62.5	6@80	3
B8-25-3d	L-ERC	25	75	62.5	6@80	3
B9-25-1d	L-ERC	25	25	62.5	6@80	3
C1-16-5d	ERC	16	80	25	6@80	3
C2-16-5d	ERC	16	80	25	6@80	3
C3-25-3d	ERC	25	75	87.5	6@80	3
D1-16-5d	ERC	16	80	67	/	3
D2-20-5d	ERC	20	100	65	/	3
E1-16-10d	ERC	16	160	67	6@80	3
E2-16-17d	ERC	16	280	67	6@80	3
E3-16-10d	L-ERC	16	160	67	6@80	3
E4-16-17d	L-ERC	16	280	67	6@80	3
F1-16-5d	ERC	16	80	67	6@80	3
F2-16-3d	ERC	16	48	67	6@80	3
F3-16-1d	ERC	16	16	67	6@80	3

**Table 4:** Test data and failure mode of specimens.

Specimen Number	$f_{cu}$ (MPa)	$f_{tu}$ (MPa)	F (kN)	$\tau_{u}$ (MPa)	S (mm)	Failure Mode
A1-16-5d	53.26	6.55	81.62	20.31	5.25	B
A2-16-3d	53.26	6.55	53.37	22.13	3.98	B
A3-16-1d	53.26	6.55	18.04	22.45	2.42	B
A4-20-5d	53.26	6.55	154.27	24.56	10.62	B
A5-20-3d	53.26	6.55	114.98	30.51	7.79	B
A6-20-1d	53.26	6.55	38.56	30.70	3.54	B
A7-25-5d	53.26	6.55	169.82	17.31	10.83	S
A8-25-3d	53.26	6.55	170.49	28.92	7.89	S
A9-25-1d	53.26	6.55	60.02	30.58	4.33	B
B1-16-5d	32.58	3.68	43.44	10.81	3.65	B
B2-16-3d	32.58	3.68	27.43	11.38	3.07	B
B3-16-1d	32.58	3.68	13.83	17.20	2.3	B
B4-20-5d	32.58	3.68	74.76	11.91	6.71	B
B5-20-3d	32.58	3.68	63.92	16.96	4.34	B
B6-20-1d	32.58	3.68	24.30	19.35	2.73	B
B7-25-5d	32.58	3.68	100.79	10.27	6.71	S
B8-25-3d	32.58	3.68	64.36	10.93	4.34	S
B9-25-1d	32.58	3.68	39.64	20.20	2.73	B
C1-16-5d	53.26	6.55	68.14	16.95	4.92	B
C2-16-5d	53.26	6.55	60.49	15.05	4.4	S
C3-25-3d	53.26	6.55	181.37	30.79	9.78	B
D1-16-5d	53.26	6.55	80.83	20.11	5.11	B
D2-20-5d	53.26	6.55	124.88	19.89	6.29	S
E1-16-10d	53.26	6.55	109.6	13.63	/	Y
E2-16-17d	53.26	6.55	118.98	8.46	/	Y
E3-16-10d	32.58	3.68	84.96	10.57	5.13	B
E4-16-17d	32.58	3.68	104.94	7.46	/	Y
F1-16-5d	53.26	6.55	61.12	15.20	5.52	B
F2-16-3d	53.26	6.55	42.51	17.65	4.19	B
F3-16-1d	53.26	6.55	14.7	18.28	3.13	B

## Data Availability

The original contributions presented in this study are included in the article. Further inquiries can be directed to the corresponding author.
